# Comparing Clinical Outcomes in Cardiac Surgical Patients Who Receive Sugammadex Versus Placebo: A Prospective Randomized Blinded Controlled Trial

**DOI:** 10.1097/CCE.0000000000001406

**Published:** 2026-04-22

**Authors:** Steven B. Greenberg, Noah Ben-Isvy, Andrew R. Locke, Nikola Dobrilovic, Rebecca Shamberg, Andrew Bochenek, Daneel Patoli, Chi Wang, Mohammed Minhaj

**Affiliations:** 1 Department of Anesthesiology, Critical Care and Pain Medicine, Endeavor Health, Evanston, IL.; 2 Chicago College of Osteopathic Medicine, Midwestern University, Downers Grove, IL.; 3 Department of Cardiothoracic Surgery, Endeavor Health, Evanston, IL.; 4 Department of Statistics and Methodology, Endeavor Health, Evanston, IL.

**Keywords:** cardiac surgery, cardiopulmonary bypass, fast-track extubation, neuromuscular monitoring, residual neuromuscular blockade

## Abstract

**OBJECTIVES:**

To compare the difference in the number of cardiopulmonary bypass surgical patients who receive sugammadex vs. placebo and who meet the Society of Thoracic Surgery early extubation quality benchmark.

**DESIGN:**

Single-center, randomized, double-blind, placebo-controlled trial.

**SETTING:**

Participants were enrolled at a single U.S. hospital between August 2023 and July 2025.

**PATIENTS:**

Seventy-four eligible cardiac surgery patients undergoing cardiopulmonary bypass with anticipated institutional fast-track extubation were enrolled; 64 were included in the analysis.

**INTERVENTIONS:**

Administration of either sugammadex or placebo 15 minutes after arrival to the ICU following cardiac surgery.

**MAIN OUTCOMES AND MEASURES:**

The primary outcome was the number of patients meeting the Society of Thoracic Surgery quality benchmark of early extubation in the sugammadex vs. placebo groups. Secondary outcomes encompassed specifics related to clinical outcomes, medication requirements, and nursing perception.

**MEASUREMENTS AND MAIN RESULTS:**

Surgery duration (*p* = 0.0004), bypass duration (*p* = 0.0177), and intraoperative blood products (*p* = 0.0003) were all increased in the sugammadex group. No difference was observed in the primary outcome and 96.9% of patients in both groups were extubated within 6 hours after surgery. However, all patients in the sugammadex group achieved a train of four greater than or equal to 0.9 before extubation compared with only 37.5% of the placebo group (*p* < 0.0001). The intraoperative dose of rocuronium (mean *p* = 0.0119; median *p* = 0.0047) was significantly increased in the sugammadex group. All additional demographics and secondary outcomes were comparable between groups.

**CONCLUSIONS:**

This trial found no difference in the number of patients who achieved the early extubation benchmark in the sugammadex vs. placebo groups; however, a significant number of patients in the placebo group had residual neuromuscular weakness as defined by quantitative neuromuscular monitoring. Further studies are required to investigate the implications of the high incidence of quantitative monitoring related weakness in this population without the use of sugammadex.

**KEY POINTS**

**Question**: Does administering sugammadex after cardiac surgery result in an increase in the number of patients who meet the Society of Thoracic Surgery (STS) quality benchmark of early extubation within 6 hours of the end of surgery compared with patients who do not receive neuromuscular blockade (NMB) reversal?

**Findings**: In this prospective randomized blinded controlled trial of 64 cardiac surgery patients undergoing cardiopulmonary bypass, no difference was found in the number of patients meeting the STS 6-hour extubation quality metric between the two study groups.

**Meaning**: Although this study did not demonstrate a correlation between sugammadex administration and the number of patients meeting the STS 6-hour extubation quality metric, sugammadex administration in cardiac surgery patients minimizes residual NMB in the ICU, measured by quantitative neuromuscular monitoring.

Extended durations of mechanical ventilation in cardiac surgical patients may increase the risk of respiratory complications, ICU and hospital length of stay (LOS), and immobility (1). Reducing the length of postoperative mechanical ventilation in this patient population can lead to improved outcomes (1–3). Therefore, the Society of Thoracic Surgery (STS) developed a quality benchmark metric defined as extubation within 6 hours of the end of primarily low risk cardiac surgery to potentially improve surgical recovery, specifically by reducing respiratory complications and ICU and hospital LOS (4–6).

The use of neuromuscular blockades (NMBs) without routine NMB reversal in cardiac surgical patients may serve as an obstacle to achieving the STS extubation benchmark goal, while also increasing the risk of complications (7). Without routine NMB reversal, NMB is typically metabolized by the liver and kidneys (8). After gradual metabolism, cardiac surgery patients are then liberated from the ventilator postoperatively in the ICU. The proposed reason for this strategy is to reduce the potential risk of rebleeding or arrhythmias due to a sympathetic response from patients. A survey among 495 cardiac anesthesiologists in the United States in 2002 suggested that only 9% of anesthesiologists routinely reverse NMB in cardiac surgery patients before extubation (9).

The lack of reversal drug use among any surgical patient population could result in residual NMB (rNMB), which is defined by the 2023 ASA practice guidelines for monitoring and antagonism of NMB as a train of four (TOF) ratio less than 0.9 (10, 11). Patients who do not meet this level of neuromuscular recovery are at risk for adverse outcomes including hypoxemia, airway obstruction, impaired swallowing function, increased risk for aspiration, prolonged LOS, postoperative respiratory complications, and need for reintubation (12, 13).

Recent literature has indicated potential advantages associated with NMB reversal, especially sugammadex, in cardiac surgery patients, including a decreased time to extubation (14–16). No present studies prospectively investigate whether sugammadex administration can increase the percentage of those elective and urgent cardiac surgical patients undergoing cardiopulmonary bypass to achieve the STS early extubation benchmark criteria by reducing the risk of rNMB and subsequently decreasing the time to extubation.

A retrospective comparison study of cardiac surgery patients from 2018 to 2021 was conducted at the same hospital system and suggested a 15% absolute difference (*p* = 0.0428) in patients who met STS early extubation criteria and over a 1-hour reduction in time to extubation (3.6 ± 2.0 vs. 4.7 ± 2.9; *p* = 0.0098) in the sugammadex vs. no reversal group (17). We hypothesize that administering sugammadex 15 minutes after arrival to the ICU after elective or urgent (higher risk) cardiac surgery will result in a statistically significant increase in the number of patients who meet the STS quality benchmark of extubation within 6 hours of the end of surgery when compared with patients who do not receive NMB reversal.

## METHODS

This prospective randomized controlled trial enrolled both elective and urgent cardiac surgery patients undergoing cardiopulmonary bypass within the Endeavor Health hospital system between August 2023 and July 2025. The study was designed and reported according to Consolidated Standards of Reporting Trials guidelines (**Supplement 1**, https://links.lww.com/CCX/B621). The study was registered with ClinicalTrials.gov under the identifier NCT05801679. It was approved by the Institutional Review Board (IRB; Endeavor Health IRB, Approval number: EH23-005, approved on July 7, 2023, titled: A Prospective Randomized Blinded Controlled Trial Comparing Clinical Outcomes in Cardiac Surgical Patients Who Receive Sugammadex vs. Placebo). All participants provided written informed consent before participating in the study and all procedures were followed in accordance with the ethical standards of the responsible committee on human experimentation (institutional or regional) and with the Helsinki Declaration of 1975. Those patients included were between 21 and 90 years old at the time of surgery and had planned institutional fast-track extubation defined as anticipated extubation within 24 hours of the end of surgery, as determined preoperatively by the cardiac surgery team (**Supplement 4**, https://links.lww.com/CCX/B621) and confirmed by the ICU team at time of handoff between the cardiac surgery team and ICU team. Those patients who had emergency cardiac surgery cases, neuromuscular disorders, received supplemental oxygen at home before surgery, had known allergies to rocuronium or sugammadex, anticipated intubation lasting more than 24 hours, history of opioid abuse, mechanical circulatory support, and end-stage renal disease requiring dialysis were excluded from enrollment. Additionally, patients who were on greater than 2 vasopressors/inotropes postoperatively or had excessive chest tube output (> 200 cc before protocol revision or > 400 mL after protocol revision) in the first hour postoperatively were withdrawn from the study. During the trial, the protocol was revised to include patients with chest tube output less than or equal to 400 mL within the first hour postoperatively, as the original limit of 200 mL proved too restrictive following the enrollment of the first four patients, two of whose outputs exceeded the 200 mL threshold (**Supplement 3**, https://links.lww.com/CCX/B621).

Intraoperative guidelines for the study included exclusive use of rocuronium as a neuromuscular blocking agent for intubation, fentanyl/sufentanil use for analgesia, and sevoflurane titrated to a Bispectral Index (Medtronic, Minneapolis, MN) of 40–60 during the surgery. Postoperatively, sugammadex or placebo was administered 15 minutes after arrival to the ICU and rNMB recovery was quantitatively monitored by using a noninvasive electromyography device, TetraGraph (Senzime AB, Uppsala, Sweden). The patient, surgical team, and ICU team were all blinded regarding the study drug and randomization was completed by the investigational pharmacy team. Subjects were randomized via a randomization list maintained by the investigational pharmacy team that was created by a random sequence generator. Those patients randomized to the placebo group received 0.9% sodium chloride instead of sugammadex, but in equal volumes. Study drug was dosed based on actual body weight, according to investigational pharmacy guidance, and consistent with sugammadex package insert recommendations (18). Upon surgical closure, a patient who had a TOF less than 2 was given sugammadex 4 mg/kg in the ICU and a TOF greater than or equal to 2–4 corresponded to a 2 mg/kg dose. Study drug dosing was based on qualitative TOF measurements performed once at beginning of surgical closure and consistent with institutional practice and sugammadex package insert recommendations (18). Postoperative TOF measurements were obtained by a quantitative neuromuscular monitor and were recorded by trained study staff at study drug administration, 5 minutes after study drug administration, and hourly thereafter until extubation.

Demographic data collection included age, sex, race, ASA status, body mass index, elective vs. urgent procedure, procedure type, surgery duration, cardiopulmonary bypass duration, number of intraoperative blood products, and number of system comorbidities (liver, kidney, neurologic, endocrinologic, respiratory, and cardiac). The primary outcome in this trial was the number of patients meeting the STS quality benchmark of extubation within 6 hours of the end of surgery in the sugammadex vs. placebo groups. The secondary outcomes in this trial consisted of comparison of time to first extubation, proportion of patients achieving a TOF ratio greater than or equal to 0.9 before extubation, ICU and hospital LOS, incidence of reintubation and postextubation pneumonia, ICU nursing perception of quality of recovery within first 24 hours (using a 5-point Likert scale), postextubation oxygen/noninvasive ventilation requirements, postextubation hypoxemic episodes, and medication consumption (including midazolam, propofol, rocuronium, and morphine milligram equivalents [MMEs]) preoperatively, intraoperatively, and postoperatively until 24 hours postextubation between the two study groups. Postextubation oxygen/noninvasive ventilation requirements were defined as any use of high-flow nasal cannula or noninvasive ventilation such as continuous positive airway pressure/bilevel positive airway pressure for respiratory support within the first 24 hours postextubation. Postextubation hypoxemic episodes were defined according to the Berlin criteria where Pao_2_/Fio_2_ ratios were approximated using previously validated peripheral oxygen saturation (Spo_2_)/Fio_2_ ratios every 6 hours for 24 hours postextubation. Spo_2_/Fio_2_ less than 235 corresponded to moderate to severe hypoxemia and 235–315 corresponded to mild hypoxemia (19, 20).

We calculated the power analysis using an estimated medium effect size of 0.72 with a two-sided alpha of 0.05. A total sample size of 64 (*n* = 32 in each group) would achieve a statistical power of 80%. Data were summarized using mean and sd or median and interquartile range for continuous variables and frequency and percentage for categorical variables. We examined data distributions and tested all variables for normality before testing the main hypothesis. We compared group differences in continuous variables using *t* test (parametric) or Mann-Whitney *U* test (nonparametric) and categorical variables using chi-square test or Fisher exact test (for frequency cells < 5). We considered *p* values of equal to or less than 0.05 statistically significant. All statistical analyses were performed using SAS 9.4 (SAS, Cary, NC).

## RESULTS

**Figure 1.**
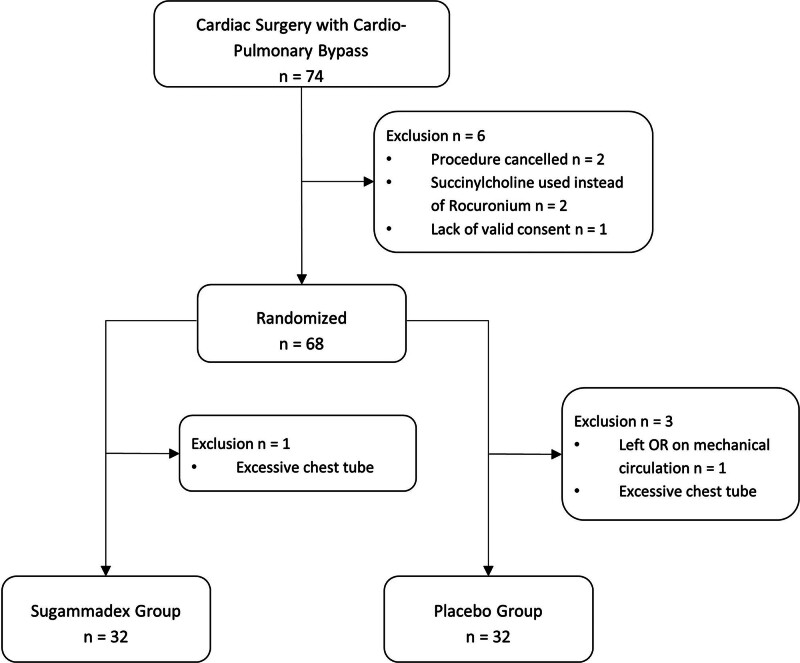
Flow diagram of randomization and exclusion of patients in the study. OR = operating room.

A total of 74 patients were enrolled in the study, and 64 patients were included in the analysis based on the previously outlined criteria. Three patients were withdrawn due to excessive chest tube output (> 200 cc before protocol revision or > 400 cc after protocol revision). Two patients were withdrawn due to canceled procedures. Two patients were withdrawn due to the use of succinylcholine instead of rocuronium for NMB. One patient was withdrawn due to requiring mechanical circulatory support after surgery, one patient was withdrawn due to lack of a valid consent form, and one patient was withdrawn due to study closure before their scheduled procedure (**Fig. 1**).

**Table 1** and **Supplement 2** (https://links.lww.com/CCX/B621) depict patient demographic characteristics. The mean cardiopulmonary bypass duration (*p* = 0.0177), the mean surgery duration (*p* = 0.0004), the median surgery duration (*p* = 0.0050), the mean intraoperative blood products (*p* = 0.0003), and the median intraoperative blood products (*p* = 0.0015) were all significantly increased in the sugammadex group vs. placebo (Table 1). There were no other significant differences in demographic characteristics between groups.

**TABLE 1.** Patient Demographics by Study Group

Demographic Variable	Total	Placebo	Sugammadex	*p*
*n* (%)	64	32 (50)	32 (50)	
Age^a^	62 (12)	61 (13)	63 (11)	0.4639
Age^b^	64 (55–72)	63 (54–72)	64 (55–74)	0.6161
Sex				0.2482
Female	16 (25.0)	10 (31.3)	6 (19.4)	
Male	48 (75.0)	22 (78.8)	26 (81.3)	
Race				0.4317
Caucasian	46 (73.0)	21 (65.6)	25 (78.1)	
Black/African American	2 (3.1)	2 (6.3)	0 (0.0)	
Asian	6 (9.4)	3 (9.4)	3 (9.4)	
Hispanic/Latino	5 (7.8)	4 (12.5)	1 (3.1)	
Other	5 (7.8)	2 (6.3)	3 (9.4)	
ASA status				0.4455
2	1 (1.6)	1 (3.1)	0 (0.0)	
3	25 (39.1)	14 (43.8)	11 (34.4)	
4	38 (59.4)	17 (53.1)	21 (65.6)	
BMI				
BMI^a^	28.8 (5.9)	28.6 (5.3)	28.9 (6.5)	0.8154
BMI^b^	27.7 (24.5–31.9)	28.9 (25.2–31.5)	26.9 (24.4–33.6)	0.9201
Intraoperative parameters				
Bypass duration (hr)^a^	2.18 (0.67)	1.98 (0.43)	2.38 (0.80)	0.0177
Bypass duration (hr)^b^	1.97 (1.73–2.47)	1.90 (1.70–2.26)	2.13 (1.76–2.97)	0.0881
Surgery duration (hr)^a^	7.20 (1.18)	6.70 (0.91)	7.70 (1.21)	0.0004
Surgery duration (hr)^b^	7.03 (6.49–7.77)	6.73 (6.17–7.37)	7.44 (6.80–8.73)	0.0050
Intraoperative blood products^a^	1.05 (1.99)	0.16 (0.45)	1.94 (2.49)	0.0003
Intraoperative blood products^b^	0 (0–1)	0 (0–0)	0 (0–4)	0.0015
Surgery type				0.4911
Elective	54 (84.4)	28 (87.5)	26 (81.3)	
Urgent	10 (15.6)	4 (12.5)	6 (18.8)	

BMI = body mass index.

a Mean (sd).

b Median (interquartile range).

**Table 2** depicts both primary and secondary outcomes. Analysis of the primary outcome of this study showed no difference between groups (*p* = 0.7540; Table 2). However, there was a statistically significant increase in the number of patients who achieved a TOF greater than or equal to 0.9 before extubation in the sugammadex vs. placebo group (*p* < 0.0001; Table 2).

**TABLE 2.** Outcome Measures by Study Group

Outcome Variable	Total	Placebo	Sugammadex	*p*
*n* (%)	64	32 (50)	32 (50)	
Society of Thoracic Surgery criteria met	62 (96.9)	31 (96.9)	31 (96.9)	0.7540
Time to extubation (hr)^a^	3.44 (2.63)	3.34 (1.22)	3.55 (3.55)	0.7435
Time to extubation (hr)^b^	3.04 (2.35–3.75)	3.19 (2.58–3.82)	2.80 (2.27–3.47)	0.2238
Final TOF				< 0.0001
≥ 0.9	44 (68.8)	12 (37.5)	32 (100.0)	
< 0.9	20 (31.3)	20 (62.5)	0 (0.0)	
Final TOF, continuous^a^	0.84 (0.29)	0.68 (0.34)	1.01 (0.06)	< 0.0001
Final TOF, continuous^b^	0.96 (0.80–1.03)	0.80 (0.46–0.97)	1.00 (0.96–1.04)	< 0.0001
Stay length				
ICU stay length (d)^a^	3.37 (3.75)	3.18 (3.13)	3.57 (4.32)	0.6822
ICU stay length (d)^b^	2.23 (1.83–3.59)	2.26 (2.03–3.27)	2.18 (1.57–3.93)	0.8150
Hospital stay length (d)^a^	6.76 (5.31)	5.88 (3.54)	7.65 (6.56)	0.1855
Hospital stay length (d)^b^	5.08 (4.30–6.44)	4.42 (4.27–5.41)	5.32 (4.34–8.00)	0.2092
Rate of reintubation				0.4921
No	63 (98.4)	32 (100.0)	31 (96.9)	
Yes	1 (1.6)	0 (0.0)	1 (3.1)	
Postextubation pneumonia				0.6128
No	61 (95.3)	31 (96.9)	30 (93.8)	
Yes	3 (4.8)	1 (3.1)	2 (6.3)	
Nursing Likert scores				
Nursing Likert scores^a^	4.21 (0.93)	4.06 (1.06)	4.34 (0.76)	0.2087
Nursing Likert scores^b^	4 (4–5)	4 (3–5)	4 (4–5)	0.3294
Postextubation hypoxemia				
Average Spo_2_/Fio_2_ over 24 hr^a^	363.22 (65.55)	373.04 (57.99)	353.39 (71.91)	
Average Spo_2_/Fio_2_ over 24 hr^b^	380.57 (312.77–416.55)	386.90 (328.92–419.52)	364.35 (312.77–405.48)	
Experienced any hypoxemia over 24 hr	43 (67.2)	21 (65.6)	22 (68.8)	0.7901
Required > 50% oxygen for more than an hour (within 24 hr)	3 (4.8)	1 (3.2)	2 (6.3)	0.8751
Required BiPAP	5 (7.9)	1 (3.2)	4 (12.5)	0.3547
Required HFNC	2 (3.2)	1 (3.2)	1 (3.1)	0.9818
Required either BiPAP or HFNC	6 (9.4)	1 (3.1)	5 (15.6)	0.1961

BiPAP = bilevel positive airway pressure, HFNC = high-flow nasal cannula, Spo_2_ = peripheral oxygen saturation, TOF = train of four.

a Mean (sd).

b Median (interquartile range).

**Table 3** depicts the consumption of midazolam, propofol, rocuronium, and MMEs preoperatively, intraoperatively, and postoperatively until 24 hours postextubation. The intraoperative (mean and median) dose of rocuronium (*p* = 0.0119, *p* = 0.0047, respectively) was significantly increased in the sugammadex group compared with the placebo group (Table 3). There were no other significant differences in secondary outcomes.

**TABLE 3.** Perioperative Midazolam, Propofol, Opioid, and Rocuronium Consumption by Study Group

Medication Variable	Total	Placebo	Sugammadex	*p*
*n* (%)	64	32 (50)	32 (50)	
Total opioid consumption (MMEs)				
Total MME^a^	437.61 (234.78)	404.19 (146.20)	471.02 (297.22)	0.2597
Total MME^b^	385 (325.5–480)	383.75 (317.5–446.25)	393.75 (329.25–428.5)	0.4273
Study drug timing				
Time between study drug and last NMB dose^a^	2.41 (0.90)	2.27 (0.08)	2.55 (0.97)	0.2009
Time between study drug and last NMB dose^b^	2.29 (1.88–2.81)	2.22 (1.8–2.63)	2.35 (1.95–3.15)	0.3178
Preoperative medications				
Preoperative midazolam^a^	0.44 (0.78)	0.41 (0.76)	0.47 (0.08)	0.7496
Preoperative midazolam^b^	0 (0–1)	0 (0–0.5)	0 (0–1)	0.7689
Preoperative propofol^a^	0 (0)	0 (0)	0 (0)	0.9999
Preoperative propofol^b^	0 (0–0)	0 (0–0)	0 (0–0)	0.9999
Preoperative MME^a^	0.47 (2.63)	0.47 (2.65)	0.47 (2.65)	0.9999
Preoperative MME^b^	0 (0–0)	0 (0–0)	0 (0–0)	0.9999
Intraoperative medications				
Intraoperative midazolam^a^	7.53 (3.92)	7.16 (4.25)	7.91 (3.58)	0.448
Intraoperative midazolam^b^	7.5 (5–10)	7 (5–10)	8 (6–10)	0.3452
Intraoperative propofol^a^	340.12 (197.73)	325.26 (141.68)	354.52 (242.52)	0.5586
Intraoperative propofol^b^	219.15 (208.62–432.09)	291.15 (218.88–425.48)	289.74 (179.62–488.41)	0.9291
Intraoperative rocuronium^a^	208.75 (59.46)	190.31 (59.86)	227.19 (53.84)	0.0119
Intraoperative rocuronium^b^	200 (170–250)	170 (145–210)	215 (195–250)	0.0047
Intraoperative MME^a^	275.74 (111.50)	260.39 (54.82)	291.09 (147.55)	0.2766
Intraoperative MME^b^	270 (225–300)	270 (225–300)	262.5 (217.5–300)	0.8665
Postoperative medications				
Postoperative midazolam^a^	0.1 (0.57)	0 (0)	0.2 (0.81)	0.1841
Postoperative midazolam^b^	0 (0–0)	0 (0–0)	0 (0–0)	0.1660
Postoperative propofol^a^	195.87 (182.16)	187.27 (167.86)	204.47 (197.76)	0.7090
Postoperative propofol^b^	137.34 (81.69–235.31)	128.48 (81.69–235.31)	144 (84.09–215.68)	0.9520
Postoperative MME^a^	161.39 (206.68)	143.33 (119.20)	179.46 (268.20)	0.4899
Postoperative MME^b^	116.75 (73.75–161)	103.75 (71.25–161.25)	130 (80–158.5)	0.4792

MME = morphine milligram equivalent, NMB = neuromuscular blockade.

a Mean (sd).

b Median (interquartile range).

## DISCUSSION

This randomized controlled trial found no difference in the number of patients meeting the STS 6-hour extubation quality metric between the two study groups. However, a statistically significant increase in the number of patients who achieved a TOF greater than or equal to 0.9 before extubation was observed in the sugammadex vs. placebo group (*p* < 0.0001). It is unclear how the TOF ratio in this study played a role in the outcomes measured.

It has been well documented in the literature that rNMB is associated with weakness, postoperative pulmonary complications, increased LOS, and decreased quality of recovery (21). While the present trial found no difference between the two study groups in terms of time to extubation, reintubation, pneumonia, LOS, postoperative MMEs, and postoperative hypoxemia as a result of increased incidence of rNMB, the study did not evaluate patient specific symptoms of rNMB such as weakness, diplopia, difficulty speaking, fatigue, and postoperative nausea and vomiting, which could impact quality of recovery (10, 22). Although the clinical signs of rNMB measured in the study were similar between groups, the significant increase in the number of patients achieving TOF greater than or equal to 0.9 in the sugammadex group suggests that patients may show clinical signs or symptoms of strength, but still have quantitative weakness that was not captured by the clinical outcomes measured in this study. In addition, the current study was not powered to detect a difference in these secondary outcomes.

Another potential explanation for not observing a significant difference in the primary and secondary outcomes studied could have been the significant increase in mean surgery and cardiopulmonary bypass times in the sugammadex vs. placebo groups. Three surgeons were involved in study procedures, and each surgeon treated an equal number of patients in both study groups. Thus, the observed differences in surgery and cardiopulmonary bypass times cannot be attributed to surgeon-specific variability. Prolongation of surgery and cardiopulmonary bypass duration has been linked to increased morbidity and mortality and may affect time to extubation from end of surgery (23). Therefore, any beneficial effect from sugammadex reversal could have been negated by the increase in surgery and cardiopulmonary bypass times. In addition, perioperative blood transfusion has also been associated with prolongation of extubation times and an increase in morbidity and mortality (24). In the present study, the mean and median intraoperative blood products (*p* = 0.0003, *p* = 0.0015, respectively) administered were significantly increased in the sugammadex group, potentially further affecting the lack of difference in primary and secondary outcomes. These findings may also indicate that the sugammadex group may have undergone more complex surgical interventions, which may have impacted extubation times and confounded the direct effects of sugammadex on recovery measures. Additionally, the mean intraoperative rocuronium (*p* = 0.0119) and the median intraoperative rocuronium (*p* = 0.0047) dosages were greater in the sugammadex compared with the placebo group. However, all subjects were administered rocuronium exclusively for maintenance of intraoperative NMB titrated to a twitch count of 2–4/4 by the end of the procedure. No patients included in the analysis received any doses of rocuronium after the TOF was calculated for sugammadex dose calculation. Thus, the difference between groups is likely not clinically significant.

Although the current study found similar times to extubation for the sugammadex and placebo groups, previous studies have suggested benefit to cardiac surgical patients receiving sugammadex with regard to decreased extubation time and hospital LOS. A prospective randomized study of 60 pediatric cardiac surgery patients suggested that the administration of sugammadex compared with neostigmine resulted in decreased time to extubation and a reduction in hospital LOS (14). However, this was a small study and lacked a placebo group in children. A retrospective study of 64 patients at a single hospital suggested that sugammadex resulted in a statistically significant reduction in mechanical ventilation time, ICU, and hospital LOS (25). However, this was a small study and did not match the patients in each group for important confounding variables such as liver/kidney disease, cardiopulmonary bypass time, and intraoperative amounts of sedative and narcotics (25). Another study by Bardia et al (16) randomized 90 elective cardiac surgical patients undergoing cardiopulmonary bypass to receive either sugammadex or placebo 30 minutes after arrival in the ICU. The sugammadex group had a statistically significant reduction in time to extubation, but no other differences were detected (16). However, this study enrolled elective healthier cardiac surgical patients (with ejection fraction > 45%) and did not use quantitative neuromuscular monitoring for sugammadex dosing or to confirm the TOF greater than 0.9 before extubation (16). The present trial enrolled real time urgent and elective cardiac surgical patients undergoing a variety of cardiac procedures, whereas Bardia et al (16) only enrolled patients undergoing coronary artery bypass grafting and/or aortic valve replacement. Additionally, the cardiopulmonary bypass time, ICU LOS, and chest tube output threshold were all lower in their study, suggesting that the current trial enrolled patients with greater variability in surgical characteristics.

Nevertheless, clinical practice pattern changes during the trial led to the decision to end the study in advance of enrolling all patients according to the present power analysis, which could account for the lack of statistical significance in the primary outcome. First, within the past several years, there has been a strong emphasis by the cardiac surgeons to extubate patients as early as possible, independent of quantitative monitoring suggestive of rNMB, as only two patients were extubated outside of the 6-hour window. Additionally, there has been a progressive increase in the clinical decision to administer sugammadex after cardiac surgery. At the time of the retrospective pilot trial, approximately 8% of cardiac surgery patients received sugammadex reversal from 2018 to 2021, and at the time of the randomized trial, approximately 50% of cardiac surgery patients received sugammadex after surgery (17). Furthermore, after moving most of the cardiac surgical cases to our newly built Cardiovascular Institute at Glenbrook Hospital, the authors estimated that sugammadex administration in cardiac surgery patients had increased above 50%, and therefore, it was not feasible to continue the randomization process.

There were several limitations associated with the present study. First, the trial was conducted at a single hospital system. Therefore, it is unclear whether the generalizability of the results to other hospital systems would be justified. Second, subject enrollment was restricted to elective and urgent cardiac surgeries on cardiopulmonary bypass with planned fast-track extubation, suggesting the results may not be applicable to all types of cardiac surgeries, including those patients that were not expected to be extubated within the first 24 hours postoperatively. Third, the relatively small sample size in the two study groups may have limited the statistical power to detect a difference in the primary outcome. However, only two patients in the entire cohort were extubated after 6 hours, so it would be unlikely to have made a difference with complete study enrollment. Fourth, data collected on rNMB complications were largely limited to clinical patient outcomes in the first 24 hours postextubation. Additional patient specific signs and symptoms including weakness, fatigue, and postoperative nausea and vomiting that may have impacted quality of recovery were not assessed, nor were any outcomes beyond the initial 24-hour period. Fifth, hypoxemia data collection was limited to every 6 hours for 24 hours postoperatively, which may have underestimated the true hypoxemia frequency as a result of undocumented transient hypoxemia. Additionally, due to the observational design and absence of detailed diagnostic evaluation, we were unable to distinguish hypoxemia related to respiratory muscle weakness from other postoperative causes. That being said, neither group significantly differed with regard to the number of hypoxemic episodes, suggesting that reversal of neuromuscular weakness from NMB via sugammadex may have less to do with this endpoint than other factors. Sixth, despite appropriately conducted randomization, the sugammadex group had increased cardiopulmonary bypass duration, surgery duration, and intraoperative blood products compared with the placebo group, which may reflect higher surgical acuity in this patient population and could have affected extubation times and other secondary outcomes studied. Seventh, although our protocol followed current ASA practice guidelines recommending extubation at a TOF ratio greater than or equal to 0.9, emerging evidence suggests that a threshold of greater than or equal to 0.95 may further reduce the risk of postextubation respiratory complications (26). Therefore, use of a 0.9 threshold may have underestimated residual neuromuscular weakness in some patients. Additionally, in several cases the bedside clinician chose to extubate control patients before they met TOF greater than or equal to 0.9 due to hemodynamic instability, agitation, or attempts at self-extubation. Strict adherence to the 0.9 cutoff in both groups could have led to longer extubation times in the control group and changes in the primary outcome. Finally, the present trial administered sugammadex 15 minutes after ICU admission, and while this was done to prevent patients from self-injury during transport to the ICU, it is possible that early reversal in the operating room may have resulted in a different outcome. Future studies should aim to study the impact of sugammadex on patient specific endpoints associated with rNMB on a larger scale across multiple hospital systems.

## CONCLUSIONS

In this blinded randomized controlled trial, no difference was observed in the number of cardiac surgery patients extubated within the STS 6-hour extubation benchmark between the sugammadex and placebo groups. Although there was an increased number of patients in the sugammadex group who achieved TOF greater than or equal to 0.9 before extubation, there were no differences observed in any of the clinical outcomes that were evaluated including postextubation hypoxemia, reintubation, postextubation pneumonia, or postoperative opioid consumption. It is possible that the lack of statistical significance of the primary outcome is due to practice pattern changes that included earlier extubation based on clinical strength rather than on quantitative monitoring data. Further studies are warranted to understand the longer-term effects of sugammadex on clinical outcomes in postoperative cardiac surgical patients and on investigating the effect of sugammadex on more subtle clinical features of rNMB seen in other patient populations.

## Supplementary Material
