# Supplementary material for: Comparing Clinical Outcomes in Cardiac Surgical Patients Who Receive Sugammadex Versus Placebo: A Prospective Randomized Blinded Controlled Trial
Source: Crit Care Explor. 2026 Apr 22;8(4):e1406. doi: 10.1097/CCE.0000000000001406 (PMC13102434; doi:10.1097/CCE.0000000000001406)

Online Supplement

Table of Contents

Supplement 1: CONSORT 2025 Checklist

Supplement 2: Patient Demographics by Study Group

Supplement 3: Study Protocol

Supplement 4: Cardiac Surgical Fast Track Weaning Protocol

Section/topic	No	CONSORT 2025 checklist item description	Reported on page no.
**Title and abstract**			
Title and structured abstract	1a	Identification as a randomised trial	1
	1b	Structured summary of the trial design, methods, results, and conclusions	2-3
**Open science**			
Trial registration	2	Name of trial registry, identifying number (with URL) and date of registration	3
Protocol and statistical analysis plan	3	Where the trial protocol and statistical analysis plan can be accessed	11
Data sharing	4	Where and how the individual de-identified participant data (including data dictionary), statistical code and any other materials can be accessed	10
Funding and conflicts of interest	5a	Sources of funding and other support (eg, supply of drugs), and role of funders in the design, conduct, analysis and reporting of the trial	10
	5b	Financial and other conflicts of interest of the manuscript authors	10
**Introduction**			
Background and rationale	6	Scientific background and rationale	4
Objectives	7	Specific objectives related to benefits and harms	4
**Methods**			
Patient and public involvement	8	Details of patient or public involvement in the design, conduct and reporting of the trial	5
Trial design	9	Description of trial design including type of trial (eg, parallel group, crossover), allocation ratio, and framework (eg, superiority, equivalence, non-inferiority, exploratory)	5
Changes to trial protocol	10	Important changes to the trial after it commenced including any outcomes or analyses that were not prespecified, with reason	5
Trial setting	11	Settings (eg, community, hospital) and locations (eg, countries, sites) where the trial was conducted	5
Eligibility criteria	12a	Eligibility criteria for participants	5
	12b	If applicable, eligibility criteria for sites and for individuals delivering the interventions (eg, surgeons, physiotherapists)	N/A
Intervention and comparator	13	Intervention and comparator with sufficient details to allow replication. If relevant, where additional materials describing the intervention and comparator (eg, intervention manual) can be accessed	5
Outcomes	14	Prespecified primary and secondary outcomes, including the specific measurement variable (eg, systolic blood pressure), analysis metric (eg, change from baseline, final value, time to event), method of aggregation (eg, median, proportion), and time point for each outcome	5-6
Harms	15	How harms were defined and assessed (eg, systematically, non-systematically)	
Sample size	16a	How sample size was determined, including all assumptions supporting the sample size calculation	6
	16b	Explanation of any interim analyses and stopping guidelines	N/A
Randomisation:			6
Sequence generation	17a	Who generated the random allocation sequence and the method used	
	17b	Type of randomisation and details of any restriction (eg, stratification, blocking and block size)	6

			Reported on page no.
Allocation concealment mechanism	18	Mechanism used to implement the random allocation sequence (eg, central computer/telephone; sequentially numbered, opaque, sealed containers), describing any steps to conceal the sequence until interventions were assigned	6
Implementation	19	Whether the personnel who enrolled and those who assigned participants to the interventions had access to the random allocation sequence	5
Blinding	20a	Who was blinded after assignment to interventions (eg, participants, care providers, outcome assessors, data analysts)	5
	20b	If blinded, how blinding was achieved and description of the similarity of interventions	5
Statistical methods	21a	Statistical methods used to compare groups for primary and secondary outcomes, including harms	6
	21b	Definition of who is included in each analysis (eg, all randomised participants), and in which group	6
	21c	How missing data were handled in the analysis	N/A
	21d	Methods for any additional analyses (eg, subgroup and sensitivity analyses), distinguishing prespecified from post hoc	N/A
**Results**			
Participant flow, including flow diagram	22a	For each group, the numbers of participants who were randomly assigned, received intended intervention, and were analysed for the primary outcome	6
	22b	For each group, losses and exclusions after randomisation, together with reasons	6
Recruitment	23a	Dates defining the periods of recruitment and follow-up for outcomes of benefits and harms	5
	23b	If relevant, why the trial ended or was stopped	8
Intervention and comparator delivery	24a	Intervention and comparator as they were actually administered (eg, where appropriate, who delivered the intervention/comparator, how participants adhered, whether they were delivered as intended (fidelity))	5
	24b	Concomitant care received during the trial for each group	5
Baseline data	25	A table showing baseline demographic and clinical characteristics for each group	14-18
Numbers analysed, outcomes and estimation	26	For each primary and secondary outcome, by group:<br>● the number of participants included in the analysis<br>● the number of participants with available data at the outcome time point<br>● result for each group, and the estimated effect size and its precision (such as 95% confidence interval)<br>● for binary outcomes, presentation of both absolute and relative effect size	6-7, 14-18
Harms	27	All harms or unintended events in each group	N/A
Ancillary analyses	28	Any other analyses performed, including subgroup and sensitivity analyses, distinguishing pre-specified from post hoc	N/A
**Discussion**			
Interpretation	29	Interpretation consistent with results, balancing benefits and harms, and considering other relevant evidence	7-9
Limitations	30	Trial limitations, addressing sources of potential bias, imprecision, generalisability, and, if relevant, multiplicity of analyses	7-9

Citation: Hopewell S, Chan AW, Collins GS, Hróbjartsson A, Moher D, Schulz KF, et al. CONSORT 2025 Statement: updated guideline for reporting randomised trials. BMJ. 2025; 388:e081123. https://dx.doi.org/10.1136/bmj-2024-081123
© 2025 Hopewell et al. This is an Open Access article distributed under the terms of the Creative Commons Attribution License (https://creativecommons.org/licenses/by/4.0/), which permits unrestricted use, distribution, and reproduction in any medium, provided the original work is properly cited.

*We strongly recommend reading this statement in conjunction with the CONSORT 2025 Explanation and Elaboration and/or the CONSORT 2025 Expanded Checklist for important clarifications on all the items. We also recommend reading relevant CONSORT extensions. See www.consort-spirit.org

Supplement 2: Patient Demographics by Study Group

Outcome Variable	Total	Placebo	Sugammadex	p-value
N (%)	64	32 (50)	32 (50)	
**Procedure Type**				0.5656
Valve Repair/Replacement	19 (29.7)	11 (34.4)	8 (25.0)	
Multi Valve Repair/Replacement	3 (4.7)	1 (3.1)	2 (6.3)	
CABG	26 (40.6)	12 (37.5)	14 (43.8)	
Valve Repair/Replacement and CABG	3 (4.7)	1 (3.1)	2 (6.3)	
Aortic Aneurysm Repair	5 (7.8)	3 (9.4)	2 (6.3)	
Redo Sternotomy	2 (3.1)	0 (0.0)	2 (6.3)	
Valve Repair/Replacement and Aortic Aneurysm Repair	3 (4.7)	3 (9.4)	0 (0.0)	
ASD	2 (3.1)	1 (3.1)	1 (3.1)	
Valve Repair/Replacement, CABG, and Aortic Aneurysm Repair	1 (1.6)	0 (0.0)	1 (3.1)	
**System Comorbidities**				
Liver	0 (0.0)	0 (0.0)	0 (0.0)	-
Kidney	4 (6.3)	2 (6.3)	2 (6.3)	0.6936
Neurologic	5 (7.8)	4 (12.5)	1 (3.1)	0.3547
Endocrinologic	21 (32.8)	10 (31.3)	11 (34.4)	0.7901
Respiratory	20 (31.3)	9 (28.1)	11 (34.4)	0.5896
Heart	63 (98.4)	32 (100.0)	31 (96.9)	0.3135
Any comorbidity	64 (100.0)	32 (100.0)	32 (100.0)	-

[a]Mean (standard deviation), [b]Median (interquartile range)

Supplement 3: Study Protocol

Subject Recruitment
Study subjects will be recruited from the Cardiac Surgical Clinic at Endeavor Health. The research team will screen all potential subjects seen at the Cardiac Surgical Clinic. Eligible subjects may be contacted in person and/or by phone to discuss the study. Informed consent can be obtained in-person in the surgeon's clinic prior to the subjects' surgery date or via an e-consent link directly from REDCap. Preferred email addresses will be confirmed prior to sending the study consent form. The study staff will document in EPIC a telephone call that will occur with patients prior to their surgery. Documentation of this call will include that the patient confirms receipt of the consent. All enrolled subjects will be enrolled only once and will be assigned a subject identification number during the consent visit that will be used on all subject documentation. The cardiac surgeon will identify those patients who he believes will meet the criteria for fast extubation within 24 hours. Those patients who have neuromuscular disorders, on home oxygen, on mechanical circulatory support or require mechanical circulatory support postoperatively, who are on > 2 vasopressors or inotropes postoperatively, have an intraoperative estimated blood loss of > 1liter excluding cell saver, difficult airway identified by anesthesia team in the operating room, or chest tube output >400cc/hr. in first hour of postoperative period will be excluded from the study after potential enrolment. All other patients will be enrolled in the fast-track limb and are anticipated to be extubated within 24 hours.

Anesthetic Management
All subjects enrolled in the study will receive the standard general anesthetic with inhaled sevoflurane to keep the bispectral index (BIS®) between 40-60. Fentanyl or sufentanil will be used for analgesia and will be up to the provider on when to administer. Induction drugs for general anesthesia will be up to the anesthesia providers. Subjects will be given rocuronium exclusively for neuromuscular blockade for intubation and maintenance of neuromuscular blockade during the operation. Upon surgical closure, the anesthesia team will measure a twitch count with the neuromuscular monitor and target that count between 2/4-4/4 at that time. Intraoperative providers will document the TOF count prior to transporting the subject to the ICU. Postoperatively, all subjects will be transferred to the ICU on propofol infusions or midazolam boluses, and dosing will be left up to the providers. After arrival to the ICU, patients will be randomized via a randomization list maintained by the investigational pharmacy team that was created by a random sequence generator. The investigator and research coordinator will then provide the anesthesia professional who is administering either placebo or sugammadex with the drug.

After the ICU signout has been completed (15 minutes after subject arrives to ICU), subjects will be administered either sugammadex (2mg/kg assuming a twitch count of 2-4/4) or placebo (0.9% NaCl) by the anesthesia provider. If the twitch count is less than 2, subjects will be given 4mg/kg of sugammadex or placebo. Five minutes after administration of the study drug, a quantitative neuromuscular monitor (Tetragraph, Senzime, Uppsala, Sweden) will be used to record the TOF ratio and compare it to baseline value. The surgical and ICU teams will be blinded to the administration of the study drug. The subjects will also be blinded to the administration of the study drug.

Subjects will be extubated when the following criteria have been met: (see Supplement 4). In addition to the weaning protocol, all subjects must meet the TOF ≥0.9 prior to extubation. The TOF monitoring will occur 5 minutes after administration of the study drug and every hour after that or when the subject has met all other standard extubation criteria according to the cardiac surgery weaning protocol.

Statistical Analysis and Sample Size Justification
Data analysis will be performed by Chi Wang, PhD. Comparison data will be analysed between groups using Student t-test for continuous variables and Chi-square test for categorical variables. For continuous endpoints, normality should be checked for a student t-test, if not normally distributed an alternative statistical method will be used**.** We anticipate that patient demographic and clinical characteristics will be similar between the two experimental groups due to random assignment. However, if we observe any differences in patient demographics and preoperative or intraoperative measures between the two groups, we will control these variables in the multivariable analysis.

Power/Sample Size

We conducted the power analysis based on our preliminary data cited above. A total of 152 patients (76 in each group) will achieve an 80% power to detect a 15%-point difference (95% vs. 80%) in proportion of patients who meet STS early extubation criteria (within 6 hours of end of surgery) between intervention and placebo groups using a two-sided alpha of 0.05. This sample size will also allow us to detect a medium effect size of d=0.46 in time to extubation between the two groups with an 80% power. All secondary outcomes will be exploratory only.

Specific Drug Supply Requirements
Merck will provide the study drug (sugammadex) to the Endeavor Health Pharmacy team as this is not current standard of care to administer sugammadex to this patient population. The pharmacy will prepare a 5cc syringe (labelled study drug) to the anesthesia professional who will administer the study drug in the ICU or the placebo (which will be saline in the same 5cc syringe). Our pharmacy will be responsible for acquiring the saline placebo for the study. Our pharmacy will also be responsible for filling individual patient containers, labeling the containers and performing the blinding of the supplies.

The investigators will be responsible for the destruction of the supplies at the study center pursuant to the ICH/GCP Guidelines, local regulations, and the investigator's institutional policies. Clinical supplies are dispensed in accordance with the protocol. The investigators will be responsible for keeping accurate records of the clinical supplies, the amount dispensed to the patients, and the disposition at the end of the study.

Adverse Experience Reporting
The PI and Co-PI will review the progress of the project and data being collected to ensure that potential adverse events are identified and responded to appropriately. We will report any adverse events to the IRB with the required documentation for reporting adverse events and serious events. Participants will have phone numbers to call the research team if they have any problems.

Unblinding
In the event that a patient becomes ineligible to continue in the study during or after surgery, the patient will be notified in person by the research staff immediately upon when the patient is conscious to understand why they are no longer in the study. The patients will continue to receive the standard of care throughout their hospital stay at Endeavor Health. Some of the reasons why patients may not continue in the study is requirement for circulatory support that was unanticipated and required after the operation, any reason why a patient might require prolonged intubation (> 24hours) such as excessive bleeding (>2L estimated blood loss), requirement for more than two vasopressors or inotropes or concern for reoperation requirement and significant hypoxemia due to fluid overload.

Protocol Amendments

One change was made to the protocol within the first year of initiation to expand the inclusion criteria. During the trial, the exclusion criteria of chest tube output ≤200ml within the first hour postoperatively was increased to ≤400ml. This change was made because the original limit of 200ml proved too restrictive following the enrollment of the first four patients, two of whose outputs exceeded the 200ml threshold.

Supplement 4: Cardiac Surgical Fast Track Weaning Protocol

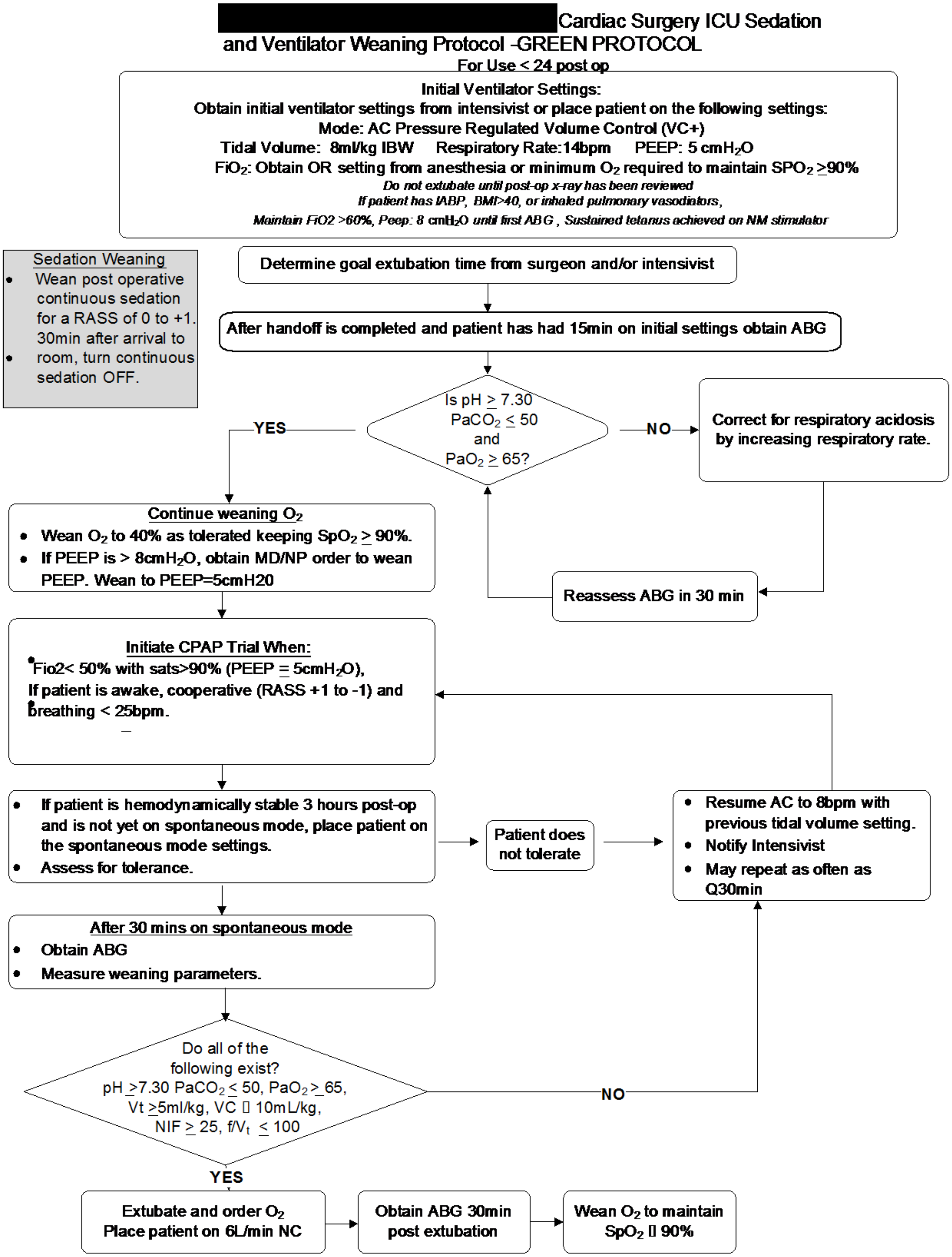

Supplement: Supplementary file 1 [file cc9-8-e1406-s001.pdf]
